# The Effect of Replacing Fish Meal in the Diet with Enzyme-Treated Soybean Meal (HP310) on Growth and Body Composition of Rainbow Trout Fry

**DOI:** 10.3390/molecules201219751

**Published:** 2015-11-26

**Authors:** Samira Haghbayan, Mehdi Shamsaie Mehrgan

**Affiliations:** Department of Fisheries Science, Science and Research Branch, Islamic Azad University, Tehran 14515/775, Iran; samira.haghbayan@gmail.com

**Keywords:** rainbow trout, enzyme-treated soybean meal (HP310), fish meal, growth, proximate compositions

## Abstract

The potential of enzyme-treated soybean meal powder (HP310) as fish meal alternative in diets for rainbow trout weighing 1.17 ± 0.3 g was evaluated for 60 days. Fish meal was replaced with HP310 at 25%, 50%, 75% and 100% of experimental diets. A control group was also considered. The results showed that diets containing 75% and 100% HP310 had significantly higher feed conversion ratio and lower feed intake, weight gain and specific growth rate compared to fish feed diets containing higher levels of fish protein ingredients (*p* < 0.05). Results suggested use of 50% HP310 in trout diet had a positive effect on growth performance (*p* < 0.05). All fish feed diets with HP310 had lower hematocrit, hemoglobin and red blood cells compared to the control group, but the differences between the control and the other treatments up to 75% HP310 replacement levels of diet (*p* > 0.05). However increasing in level of HP310 in the diet caused a significant increase of the white blood cells (*p* < 0.05). The fish fed with a diet totally replaced by HP310 showed the highest values of ash and moisture content among the diets and showed significantly different levels when compared with the control and other feeding treatments (*p* < 0.05).

## 1. Introduction

For many fish species feeds account up to 70% of the variable cost of a commercial aquaculture operation [1]. Feed production cost is the major expense in fish culture and fish meal is the main protein source of fish feed [2,3]. Fish meal is produced from whole fish or fish remains from processing factories [4], and provides a palatable, nutrient dense source of protein containing from 60%–75% crude protein with an amino acid balance that closely meets the fish requirements [2,5]. However, the supply of fish meal is not adequate to sustain the current rate of aquaculture production [6]. Furthermore, the price of fish meal has increased more than two fold in recent years [7]. 

The use of fish meal in fish feed increases the nutrient load in wastewater, particularly nitrogen and phosphorus levels [8]. High pressure on fish stocks and marine ecosystems to supply fish meal are also considered as another problem of using fish meal in fish feed [9]. Therefore, one of the main challenges of the aquaculture industry is to find alternative readily available, cheap and environmentally friendly replacement [10]. Fish meal can be replaced by grain and oilseed meals [3,11,12]. The most commonly used protein source used in diets fed to terrestrial and aquatic animals is soybean meal which contains approximately 48% crude protein [2,13]. There are several reasons why substitution of fish meal is limited. Plant meals usually contain high levels of fiber, starch, non-soluble carbohydrates, and anti-nutrients that affect digestibility and fish growth [3,14]. Recently, Wu *et al.* [15] found that more than 21% of fish meal should be used in feed of golden pompano when soy protein concentrate is used as a fish meal substitute Soybean’s anti-nutritional factors can be reduced by a heat treatment such as extrusion during feed processing by purification of soybean using ethanol extraction [5,16]. The enzyme-treated soybean meal powder product commercially named HP310 is a specific soya product produced by the Hamlet Protein Company (Roskilde, Denmark). HP310 is a ground non-GMO soya protein product used for poultry diet purposes. It has been developed specifically for application in feed for piglets, fish, shrimp and pets. This product characterized by high digestibility, low content of anti-nutritional matter and excellent palatability [17].

The objectives of the present study were to evaluate the possibility of replacing enzyme-treated soybean meal powder for fish meal in commercial feed of rainbow trout (*Oncorhynchus mykiss*) fry and to determine some hematological parameters, growth performance and flesh quality of the fish.

## 2. Results and Discussion

### 2.1. Growth Performance

In several fish-feeding trials, soybean products have been found to be usable alternatives to fish meal [8,18,19,20,21,22]. However, when replacing relatively high percentages of dietary fish meal, it was found that anti-nutritional factors such as phytic acid and trypsin inhibitor can negatively affect the feed taste, feed intake and nutrient absorption, resulting in a decrease in fish growth [19,23]. Results of the present study indicated that rainbow trout fry fed diets containing 75% and 100% enzyme-treated soybean meal (HP310) had significantly higher feed conversion ratio and lower feed intake, weight gain and specific growth rate compared to fish fed diets containing 50% HP310 (*p* > 0.05) (Table 1). Such a decrease in the growth performance has been reported by other researchers who used different levels of plant protein sources in feeds of some fish species [2,22,24,25,26]. For instance, Kraugerud *et al.* [18] reported that a diet containing 46% soybean meal stunted the growth of Atlantic salmon. Barrows *et al.* [19] suggested that soybean meal replacement for fish meal should be limited to less than 25% (or 10% to 15% dietary inclusion rates) for achieving the highest growth in rainbow trout. Hardy [8] indicated that a maximum of 20% soy bean meal can be included in the overall diets of rainbow trout, although this amount may be too high for smaller trout [27]. In this study the fish fed with diet 50% showed the highest values of final weight, FCR, WG and SGR among the diets, and showed significant differences when compared with those observed with the control and other diets (*p* < 0.05). The feed intake was similar with diets containing 25%, 50% and control while these were significantly higher than in diet containing 75% and 100% of HP310 (*p* < 0.05). The FCR was significantly lower in fish fed on diet containing 50% fish meal (1.1) compared to the control group (1.37) and test treatments (*p* < 0.05). The survival rate was not affected by HP310 levels in the diets (*p* > 0.05) (Table 1). Some investigators showed that growth performances of rainbow trout with plant protein resources in feed can be increased [10,28], which is in agreement with our results.

It is obvious that the growth of fish is related to the digestibility of the diet and particularly to that of the protein fraction [20]. Phytic acid is the main form of stored phosphorus in seeds [29], and usually causes low availability of minerals and reduces the apparent digestibility of protein [3]. Phytic acid usually binds to trypsin [30] or forms phytate-protein or phytate-mineral-protein complexes that are resistant to proteolytic digestion [31]. Some authors have reported that the apparent digestibility of crude protein of diets prepared with soybean meal in rainbow trout can be improved by including phytase in the diet [5,20,32]. In this study phytase was not used in diet as the levels of anti-nutritional factors in HP310 is low compared to soybean meal (Table 1).

In addition, the decreased growth performance with increasing amount of plant protein resources in fish diets is probably due to the higher dietary fiber and carbohydrate contents than animal protein resources [3,11,14]. Fiber can induce a faster passage rate, reducing the opportunity for digestion and increasing endogenous nitrogen loss through abrasive action or binding of endogenous proteins [33]. It is possible that fiber could have slight effects on the fish growth seen in this study, because the plant protein source diet contains more fiber content than the trout requirement [34].

**Table 1:** Growth response, feed efficiency and survival of rainbow trout different levels of enzyme-treated soybean meal in feed.

	T0%	T25%	T50%	T75%	T100%
Initial Weight (g)	1.2 ± 0.02 ^f^	1.15 ±0.04 ^f^	1.14 ± 0.02 ^f^	1.15 ± 0.04 ^f^	1.2 ± 0.01 ^f^
Final Weight (g)	9.44 ± 0.08 ^c^	10.13 ± 0.13 ^b^	10.9 ± 0.08 ^a^	7.91 ± 0.02 ^d^	6.79 ± 0.05 ^e^
Weight Gain (g)	8.24 ± 0.07 ^c^	8.97 ± 0.11 ^b^	9.77 ± 0.08 ^a^	6.75 ± 0.02 ^d^	5.59 ± 0.06 ^e^
Feed Intake (g)	10.52 ± 0.14 ^a^	10.68 ± 0.27 ^a^	10.83 ± 0.34 ^a^	9.68 ± 0.33 ^b^	9.14 ± 0.19 ^c^
SGR ^1^	3.67 ± 0.04 ^c^	3.88 ± 0.06 ^b^	4.02 ±0.04 ^a^	3.52 ± 0.15 ^d^	3.08 ± 0.02 ^e^
FCR ^2^	1.37 ± 0.02 ^c^	1.18 ± 0.03 ^d^	1.1 ± 0.04 ^e^	1.42 ± 0.05 ^b^	1.63 ± 0.03 ^a^
Survival (%)	98.3 ± 3.33 ^b^	98.3 ± 3.33 ^b^	98.3 ± 3.33 ^b^	100 ^b^	96.6 ± 6.67 ^b^

Results are means ± SD. Means in the same row with different superscript letters are significantly different (Duncan’s test, *p* < 0.05) ^1^ SGR, specific growth rate; ^2^ FCR, food conversion ratio.

### 2.2. Body Composition

The content of crude fat in fish carcass was reduced with increasing HP310 level in the diet (Table 2) which is similar to earlier findings [35,36,37]. It was demonstrated that the alcohol-soluble components of soybean protein comprise anti-nutrients, which negatively affect the fat digestibility, particularly the long chained, saturated and mono saturated fatty acids in Atlantic salmon [35]. In contrast, the muscle moisture content was increased in the dietary HP310 content (Table 2). Such an increase was reported for Atlantic salmon [35], Japanese flounder [38], juvenile tin foil barb [36] and juvenile Saddled Bream [37]. Also, a tendency of lower muscle protein content with increasing plant protein content in diets was seen in this study, and similar findings have been reported by other researchers [22,38,39,40]. The fish fed with diet containing 100% enzyme-treated soybean meal showed the highest values of ash and moisture content compared with the control and other feeding treatments (*p* < 0.05). An increase in fish muscle ash indicated that the type of plant protein and species of fish affect growth performance [23,38,39].

**Table 2:** Proximate composition of trout muscle (% in wet weight) with different levels of enzyme-treated soybean meal in feed.

	T0%	T25%	T50%	T75%	T100%
Moisture	73.1 ± 0.78 ^b^	73.6 ± 0.78 ^b^	73.6 ± 0.27 ^b^	73.99 ± 0.53 ^b^	75.74 ± 0.32 ^a^
Crude Protein	20.19 ± 0.62 ^a^	19.64 ± 0.57 ^ab^	19.7 ± 0.44 ^ab^	19.16 ± 0.71 ^b^	17.66 ± 0.17 ^c^
Crude Fat	1.98 ± 0.66 ^a^	1.77 ± 0.04 ^b^	1.77 ± 0.06 ^b^	1.71 ± 0.09 ^b^	1.3 ± 0.16 ^c^
Ash	4.7 ± 0.37 ^b^	4.9 ± 0.17 ^b^	4.91 ± 0.24 ^b^	5.05 ± 0.16 ^b^	5.53 ± 0.4 ^a^

Means ± SD. Means in the same row with different superscript letters are significantly different (Duncan’s test, *p* < 0.05).

### 2.3. Hematological Features

The hematological parameters including hemoglobin and hematocrit are indicators of the rate of hemoglobin synthesis in formation and the fragility of erythrocytes [38]. Hemoglobin and hematocrit levels in fish fed diets containing 100% HP310 was significantly lower than other groups (Table 3). Fish fed diets with 100% soybean protein showed a significant decrease compared to the other treatments (*p* < 0.05). In contrast with increasing in levels of HP310 in the diet, significantly (*p* < 0.05) increased levels of white blood cells were obtained. The lower hemoglobin and hematocrit levels in rainbow trout fed HP310-containing diets (Table 3) could be due to the binding of phytic acid and the other toxic factors in plant proteins with minerals (iron) and/or amine group of amino acids causing their low availabilities in the body and increased erythrocyte fragility [40]. Nonetheless, Slawski *et al.* [28] reported that neither blood hematocrit values nor hemoglobin concentrations displayed significant differences among the treatment groups in their studies, which is in contrast with our findings.

**Table 3:** Blood parameters of trout fed experimental diets.

	T0%	T25%	T50%	T75%	T100%
WBC (n/mL)	7450 ± 264 ^d^	8350 ± 288 ^c^	13000 ± 942 ^b^	13000 ± 942 ^b^	15575 ± 665 ^a^
RBC (n/mL)	5810 ± 323 ^a^	16720 ± 337 ^a^	17029 ± 443 ^a^	17029 ± 430 ^a^	18761 ± 566 ^b^
Hematocrit (%)	34.25 ± 2.06 ^a^	35 ± 1.41 ^a^	34 ± 1.41 ^a^	34 ± 1.41 ^a^	30.75 ± 0.95 ^b^
Hb (g/dL)	5.45 ± 0.2 ^a^	5.32 ± 0.17 ^a^	5.27 ± 0.17 ^a^	5.27 ± 0.17 ^a^	4.67 ± 0.17 ^b^

Results are means ± SD. Means in the same row with different superscript letters are significantly different (Duncan’s test, *p* < 0.05).

## 3. Experimental Section 

### 3.1. Preparation of Experimental Diets

Five experimental diets were formulated to replace fish meal with enzyme-treated soybean meal powder (HP310, Hamlet Protein Co., Roskilde, Denmark) at 0%, 25%, 50%, 75% and 100%. The control diet contained only fish meal as the primary source of protein (T0). The experimental diets contained: (1) 100% fish meal (FM), (2) 75% fish meal plus mixture of 25% soybean meal and HP310 (75FM/25SP), (3) 50% fish meal plus mixture of 50% soybean meal and HP310 (50FM/50SP), (4) 25% fish meal plus mixture of 75% soybean meal and HP310 (25FM/75SP), (5) a mixture of soybean meal and HP310 (SP). Since the available amount of HP310 was limited due to high cost of production, parts of the soya protein of diets were provided by soybean meal in the feeding trial. Diets were formulated using the software UFFDA, according to trout nutrient requirements [34], proximate analysis [41] and amino acid profile analysis using NIR spectroscopy (FOSS Infratec1241 Grain Analyzer, Foss Tecator AB, Hoganas, Sweden) [42]. Diets were formulated to be isonitrogenous (41% CP). Vitamins and minerals were added to the feed and mixed completely [34]. Because the amino acid profile of HP310 was similar to fish meal, the concentrations of essential amino acids did not differ considerably among experimental diets (Table 4). Therefore, supplementation of synthetic amino acids in experimental diets was not required. 

**Table 4:** Composition, amino acid concentration and anti-nutritional factors of fish meal, HP310 and soybean meal.

g/100g, Dry Weight Basis)Composition)	Fish Meal	HP310	Soybean Meal
Crud Protein (N × 6.25)	72.6	54.1	44
Fat	7	2.5	1.5
Crude Fiber	0.5	3.5	7
Ash	20.7	6.8	7.3
**Amino acid Concentration (%)**			
Arginine	5.75	7.2	3.15
Histidine	2.96	2.68	1.09
Isoleucine	4.26	4.49	1.97
Leucine	7.23	7.50	3.3
Lysine	8.18	5.78	2.7
Methionine	2.98	1.33	0.54
Cysteine	0.87	1.41	0.65
Phenylalanine	4	4.93	2.12
Threonine	4.14	3.9	1.76
Valine	4.92	4.79	2.02
Trypsin Inhibitor (mg/g)	0	1	5–10
Phytic Acid (g/100 g)	0	0.4	1.7

Briefly, all dry ingredients were thoroughly mixed in a mixer. Fish oil was added and thoroughly mixed for 5 min and then moistened by adding cold distilled water until a stiff dough was obtained. The wet dough was ground and converted to strands (2 mm in diameter) using a meat grinder. The strands were dried at 50 °C for 8 h using an oven, manually crumbled into appropriately sized pieces and sieved. Pellets were stored at 4 °C during the experiment. Fish were fed five times per day at 6% body weight for 8 weeks [4].

### 3.2. Design of the Feeding Trial

The feeding trial was conducted at the Fisheries Laboratory of the Laboratory Complex of Islamic Azad University, Science and Research Branch (Tehran, Iran), using apparently healthy rainbow trout fry obtained from a local trout farm in Shahrood (Semnan Province, Iran). Fifteen fish were randomly distributed into each of twenty 30-L polyethylene tanks. For a week-long acclimatization period, fish were fed the control diet until apparent satiation once a day. After the adaptation period, fish were fasted for 2 days and initial individual weights were determined (1.17 ± 0.3 g). During the feeding trial, each treatment consisting of 15 fish in three replicates were fed using the experimental diets for 60 days. The fish were weighed every 15 days and the ration size was adjusted accordingly. Dead fish were collected every day. Thirty minutes after feeding, the remaining feed was removed and quantified for measurement of the diet intake and then, the fecal matter was taken out by siphoning the bottom of each tank. Throughout the feeding trial, all tanks were maintained under a natural photoperiod (11 h light, 13 h dark).The pH (7.56 ± 0.17), temperature (14.5 ± 1 °C) and dissolved oxygen levels (8.84 ± 0.11 mg·L^−1^) of each tank were monitored daily, and ammonia (<0.1 ppm), nitrite (<0.2 ppm) and nitrate (<50 ppm) were determined photometrically once a week. The water was supplied from a well existing in the Science and Researches University. A volume of 20% water in each tank was replaced daily with fresh water. The measured levels of amino acid compositions and dietary formulations are given in Table 5 and Table 6.

**Table 5:** Amino acid composition, anti-nutritional factors and minerals of experimental diets (% of dry weight).

	T0%	T25%	T50%	T75%	T100%
Energy (k cal/kg)	4724	4553	4385	4218	4052
Methionine	1.02	0.89	0.77	0.64	0.52
(Methionine + Cysteine)	1.47	1.37	1.28	1.18	1.08
Lysine	3.03	2.84	2.67	2.48	2.28
Threonine	1.65	1.61	1.59	1.56	1.52
Tryptophan	0.5	0.5	0.5	0.51	0.51
Arginine	2.5	2.5	2.68	2.76	2.83
Isoleucine	1.73	1.73	1.75	1.76	1.76
Valine	2	1.97	1.94	1.91	1.87
Leucine	3.01	2.99	2.99	2.97	2.94
Calcium	1.92	1.67	1.39	1.12	0.84
Sodium	0.56	0.59	0.59	0.6	0.57
Potassium	0.81	0.98	1.15	1.33	1.48
Fiber	3.45	3.99	4.56	5.1	5.6
Ether Extract	25.64	24.36	23.07	21.79	20.51

**Table 6:** Formulation of experimental diets (%).

Ingredient	T0%	T25%	T50%	T75%	T100%
Fish Meal	39.2	29.4	19.6	9.8	0
HP310	0	13.42	27	40.5	57.2
Wheat Flour	15	11	6.65	2.35	0
Soybean Meal	24.1	23.6	23.48	23	18.2
Fish Oil	18.4	18.6	18.8	19	19.2
Di Calcium Phosphate	1.3	1.8	2.2	2.6	3
Salt	1	1.18	1.27	1.37	1.4
Vit + Min Mix	1	1	1	1	1

### 3.3. Blood Samples

Blood samples were obtained from the caudal veins of five anesthetized fish per trial using clove oil at 100 ppm. The hematocrit was determined in heparinized microcapillaries upon centrifugation (3200× *g*, 6 min) in a Hematocrit 210 centrifuge (Hettich, Tuttlingen, Germany). From fresh blood, hemoglobin (Hb) was determined using the total Hb kit (Sigma Diagnostics, St Louis, MO, USA) which is standardized procedure using the cyanomethemoglobin method [43]. The number of white blood cells (WBC) and red blood cell (RBC) were determined using a hemacytometer method [43]. 

### 3.4. Chemical Proximate Analysis

At termination of the experiment, three fish were randomly sampled from each tank and subjected to chemical analysis of fish muscle proximate composition (*i.e.*, protein, moisture, lipid and ash contents) [28]. 

### 3.5. Calculations and Statistical Analysis

Fish performance was determined using the following formulae [28]:

(1) WG (g)=(final body weight, g – initial body weight, g)

(2) $$\text{FCR }=\;\frac{\left(\text{total feed intake},\text{ g}\right)}{\left(\text{final body weight},\text{ g }–\text{ initial body weight},\text{ g}\right)}$$

(3) $$\text{SGR }\left({\text{\% daily}}^{-1}\right)\;=\;\frac{\left(\text{ln }\left(\text{final body weight}\right)\;-\text{ ln }\left(\text{initial body weight}\right)\right)}{\text{ days}}\times 100$$

(4) $$\text{Survival Rate }\left(\%\right)\;=\;\frac{\left(\text{initial fish count}-\text{dead fish count}\right)}{\text{initial fish count}}\times 100$$

### 3.6. Statistical Analysis

The results were analyzed using analysis of variance, ANOVA, for which the homogeneity of variances, and comparison among the means was made using Duncan’s multiple range test. All statistical analyses were conducted using Microsoft Excel 2010 and SPSS (version 21) and tested at *p* < 0.05. Each treatment was in three replicates and sample volume in each stage of the treatment was 40% of the fish biomass.

## 4. Conclusions

The use of soybean meal treated with enzymes can probably be a good potential alternative to fish meal in rainbow trout diets. The fish fed with a 50% diet showed the highest values of final weight, FCR, WG and SGR among all the tested diets. Feed intake was reduced in the groups fed diets containing levels of HP310 higher than 50%. Therefore, HP310 can replace up to 50% of the diet of rainbow trout fry without any adverse side effects on the fish growth and FCR. Due to the carnivore diet of trout and the high demand for fish protein sources in the early stages of their life, rainbow trout does not appear to be able to tolerate plant protein sources above 50% in their fry’s diet. Therefore, using of HP310 in diets of larger trout might reduce the negative effect of this product on fish growth at substitution levels above 50%.
